# Hematochezia caused by eosinophilic proctocolitis in a newborn before oral feeding: a case report

**DOI:** 10.1186/s13256-017-1318-z

**Published:** 2017-06-16

**Authors:** Marie-Julie Debuf, Tania Claeys, Jean-Philippe Stalens, Luc Cornette

**Affiliations:** 1 Department of Pediatrics, Université Catholique de Louvain, Brussels, Belgium; 2 Department of Pediatrics, AZ Sint-Jan Brugge-Oostende, Bruges, Belgium; 3 Department of Pediatrics, Centre Hospitalier de Wallonie Picarde, Tournai, Belgium; 4 Department of Neonatology, AZ Sint-Jan Brugge-Oostende, Bruges, Belgium

**Keywords:** Hematochezia, Transient eosinophilic colitis, Newborn baby, Gut

## Abstract

**Background:**

Hematochezia is a frequent symptom in early infancy. However, it occurs very rarely within the immediate neonatal period, and its occurrence before any oral intake is particularly rare. Because of the “congenital” presentation of hematochezia in our patient, we initially considered our case to be a non-classical, potentially severe type of food protein-induced allergic proctocolitis. This diagnosis needs to be confirmed by an abnormal oral challenge test once the hematochezia has disappeared. If such a challenge cannot demonstrate an allergic origin, then the etiology of the hematochezia could be a neonatal transient eosinophilic colitis. Only two similar cases have been described so far.

**Case presentation:**

We report the case of a black baby boy of African origin born at 36 weeks 5 days of gestational age who presented with massive hematochezia immediately after birth. A rectosigmoidoscopy revealed a severe inflammation associated with diffuse eosinophilic infiltration on biopsy. His clinical outcome was favorable after introduction of an amino acid formula diet. We initially considered our case to be a non-classical, potentially severe type of food protein-induced allergic proctocolitis but reintroduction of standard formula milk at the age of 3 months was successful. So, our patient is the first newborn in Europe who fits the diagnosis of “neonatal transient eosinophilic colitis.”

**Conclusions:**

We discuss the possible etiology of “congenital” eosinophilic inflammation of the distal colon and conclude that hematochezia in well-looking neonates, in the absence of negative challenge tests later on, is more likely to be a neonatal transient eosinophilic colitis than an allergic proctocolitis. This new entity could be more frequent than previously thought, changing our medical care strategies for this kind of neonatal symptom.

## Background

Hematochezia or rectal bleeding in infancy can be the initial symptom of severely pathological conditions such as necrotizing enterocolitis, infectious colitis (*Salmonella*, *Shigella*, *Campylobacter*, *Yersinia*, or parasites), volvulus, and intussusception [1]. Hematochezia in infancy together with systemic manifestations such as profuse, repetitive vomiting and lethargy, often with diarrhea, can also suggest food protein-induced enterocolitis syndrome.

In a well-looking newborn baby, hematochezia mostly results from either swallowing maternal blood at the time of birth, perianal dermatitis, or anal fissure after the first passage of meconium. More rarely, it may be caused by a coagulation disorder, vitamin K deficiency, or food protein-induced allergic proctocolitis (FPIAP). FPIAP has been described as the trigger of hematochezia in well-looking neonates, in the absence of any of the other possible causes of early rectal blood loss. However, here we describe a case of a clinically well newborn baby who presented with significant hematochezia (in the absence of blood-stained amniotic liquor) within the first minutes after birth and thus before any postnatal contact with oral feeds.

## Case presentation

**Fig. 1:**
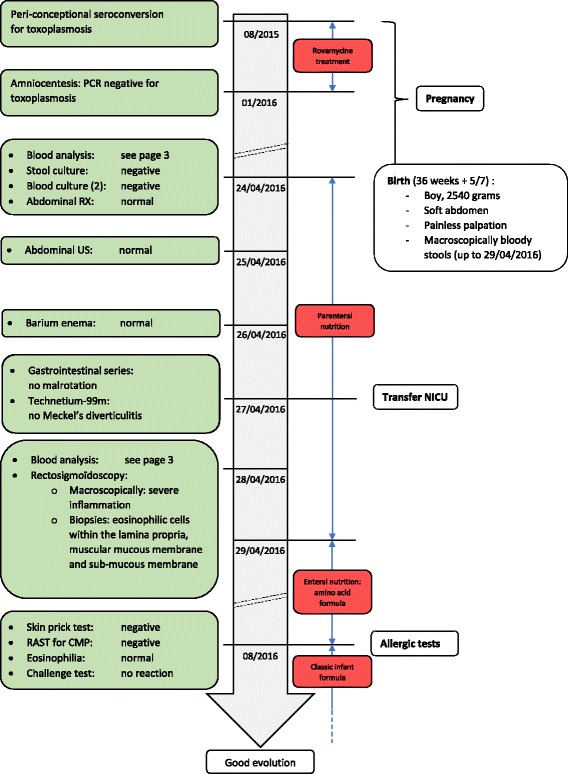
Timeline of interventions and outcomes. *CMP* cow’s milk protein, *NICU* neonatal intensive care unit, *PCR* polymerase chain reaction, *RAST* radioallergosorbent, *RX* radiography, *US* ultrasound

A black baby boy of African origin was born at 36 weeks 5 days of gestational age by vaginal delivery. He is the second child of a 24-year-old Beninese woman with no particular medical history. There was no known allergy in mother, father, or first child. The pregnancy was marked by a periconceptional seroconversion for toxoplasmosis, treated with Rovamycine (spiramycin) until obtaining a negative polymerase chain reaction (PCR) by amniocentesis (timeline shown in Fig. 1). The smear sample for Group B *Streptococcus* culture performed at 35 weeks was negative and membranes ruptured less than 12 hours before birth. The amniotic fluid was meconium stained but not bloody. The delivery was uneventful and no postpartum hemorrhage was observed.

His birth weight was 2540 grams (25th percentile on Fenton growth charts) and Apgar scores were 9 and 10 (1 and 5 minutes). A few minutes after birth, before any oral intake, he presented with macroscopically bloody stools. His abdomen was soft and painless on palpation and his anal region was normal on inspection. Blood analysis showed a hemoglobin level of 12.9 g/dL (normal values 13.5 to 19.5), platelets 133,000/μL (normal values 168,000 to 411,000), white cell count 24,930/μL (normal values 9000 to 30,000) with 13% (normal values <6) of eosinophils 3241/μL (normal values <300), C-reactive protein 5 mg/L (normal values <2.9), low immunoglobulin (Ig) E levels (normal values <2.0 U/ml), partial thromboplastin time 28.4 seconds (normal values 20 to 30), prothrombin time 17.6 seconds (normal values <21), fibrinogen 245 mg/dL (normal values 200 to 400), and international normalized ratio (INR) <1. On day 4 his hemoglobin level fell to 8.6 g/dL and his white cell count was 26,900/μL including 32% eosinophils (8508/μL). His C-reactive protein remained low (<2.9 mg/L). Stool cultures (bacterial, viral, and parasitic pathogens) as well as two blood cultures remained negative. A plain abdominal radiography, an abdominal ultrasound, and a barium enema did not demonstrate any abnormality. Upper gastrointestinal series excluded malrotation and a technetium-99m examination ruled out Meckel’s diverticulitis.

**Fig. 2:**
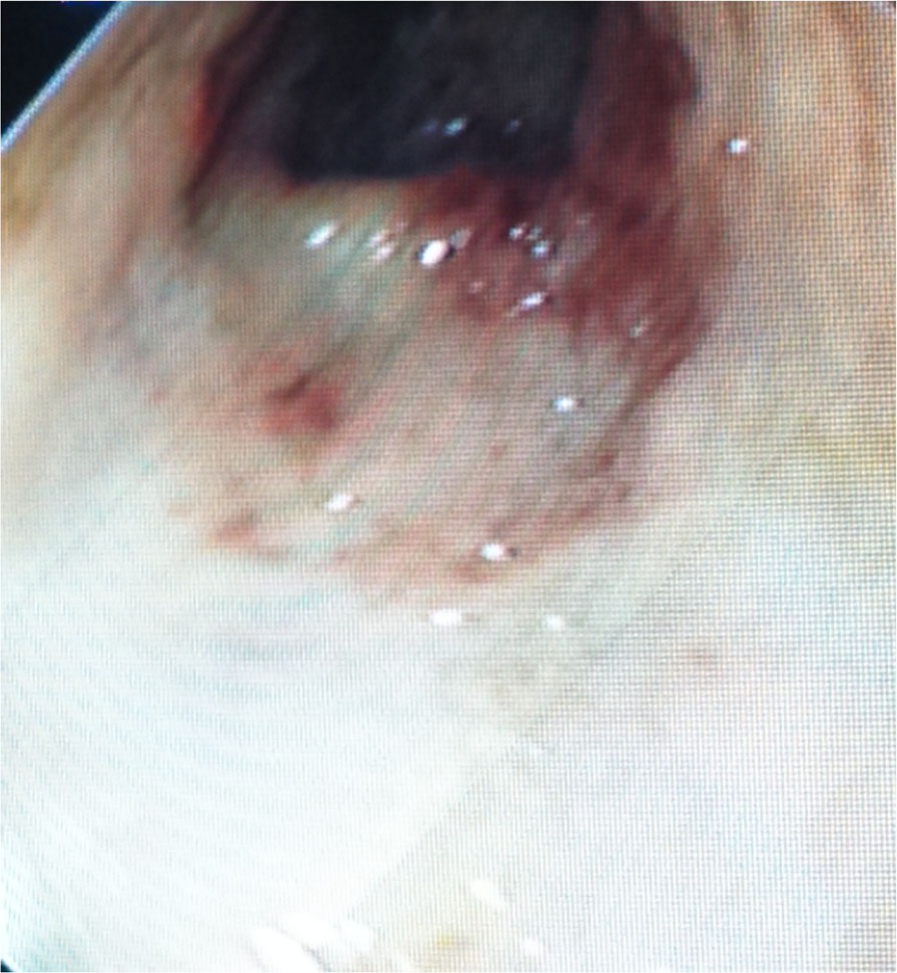
Rectosigmoidoscopy on day 4: View at 2 cm of the anal margin showing a friable, edematous colonic mucosa with nodular lymphoid hyperplasia

Oral feeds were discontinued on day 0 and he was administered fluids intravenously. Although the amount of rectal blood loss slowly decreased, the hematochezia persisted during the first days of life. Hence, a rectosigmoidoscopy was carried out on day 4 and revealed a macroscopically severe inflammation from the anal margin up to 7 cm (Fig. 2), fading out more proximally to a normal-appearing colon.

**Fig. 3:**
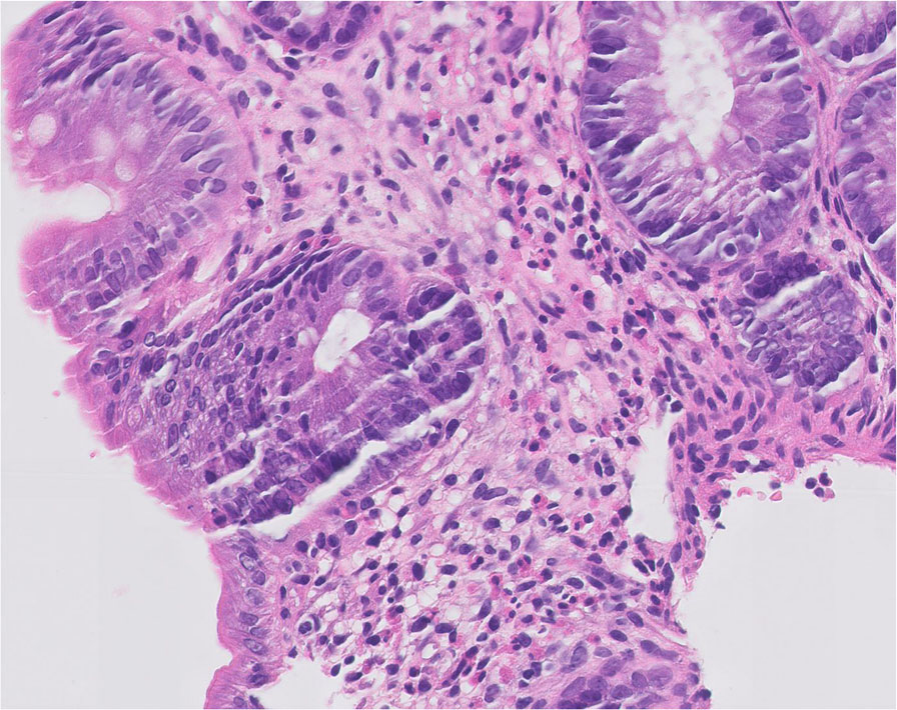
Intestinal biopsy: Biopsy taken on day 4, showing diffuse and massive infiltration of eosinophils in the lamina propria (hematoxylin and eosin staining)

All three biopsies taken (8, 13, and 17 cm from his anal sphincter) showed numerous eosinophilic cells within the lamina propria, the muscular mucous membrane, and the sub-mucous membrane (Fig. 3). No viral inclusions, granulomas, or parasites were detected.

On day 5, he was started on an orally administered amino acid formula, as a severe cow’s milk protein (CMP) allergy was suspected.

At the age of 3 months, allergy testing by means of skin prick tests and radioallergosorbent tests (RAST) for CMP (alpha-lactalbumin, beta-lactoglobulin, casein) were negative. Eosinophilia was normalized (<5% of leukocytes). He was briefly admitted to hospital for a medically supervised challenge test, reintroducing increasing quantities of standard formula containing CMP over 2 days. No reaction was observed. He was discharged with a classic baby formula.

## Discussion

This is an unusual case of a healthy newborn baby who presented with a significant hematochezia during the first hour of life, before any feeding, and in the absence of maternal blood ingestion or anal fissure(s). A rectosigmoidoscopy with biopsies revealed a significant infiltration of eosinophilic cells in all intestinal layers, highly suggestive of allergic proctocolitis (FPIAP). Our case is, however, different from the classically described entity of FPIAP, in which the allergic proctocolitis is commonly induced by postnatal and direct exposure to specific food proteins.

Classical FPIAP is a benign condition with a usual onset during the first 2 months of life due to a non-IgE-mediated immune reaction toward ingested proteins, such as cow’s milk, soy, egg, rice, fish, and wheat [2]. CMP is implicated in almost all cases [3–6]. The blood loss in FPIAP is typically modest but can occasionally result in anemia and hypoalbuminemia. The prevalence of FPIAP is around 1.5% and approximately 60% of the cases are observed in exclusively breastfed babies [5, 7, 8]. Additional investigations are not deemed necessary in a baby with a typical history and the diagnosis needs to be confirmed or excluded by an allergen elimination procedure [4, 6, 9]. Histopathologically, these eosinophils release multiple cytotoxic agents (eosinophil peroxidase, eosinophil cationic protein, major basic protein) and the immunomodulatory cytokines interleukin (IL)-1, IL-6, and tumor necrosis factor-alpha (TNF-alpha), resulting in local inflammation and tissue damage. Withdrawal of the culprit protein trigger(s) usually results in the resolution of symptoms. Bloody diarrhea typically disappears within 72 to 96 hours, but endoscopic and histologic healing can take several weeks [5]. Most babies tolerate subsequent reintroduction of the protein by the age of 1 to 3 years [4].

A diagnosis of FPIAP is rare in newborn babies presenting with hematochezia during the first week of life. Hwang and Hong reported a population of 16 well-looking patients with hematochezia during the first 6 weeks of life [10]. They were breastfed or on a classical baby formula: 88% (*n*=14) improved spontaneously or no longer demonstrated hematochezia after a challenge test. Hence, only 12% (*n*=2) of this small population presenting with hematochezia before 2 months of age were diagnosed as having classical FPIAP. Arvola *et al.* analyzed 40 children with hematochezia between 4 weeks and 6 months of age [9]. CMP allergy was diagnosed by provocation testing in 17.5% (*n*=7), showing recurrence of rectal bleeding, severe eczema, or another unambiguous adverse reaction after reintroduction of CMP.

Because of a “congenital” presentation of hematochezia, we initially considered our case to be a non-classical, potentially severe type of FPIAP, possibly to CMP after *in utero* sensitization. Such maternofetal sensitization can occur through active transplacental transport of allergens or by fetal uptake of allergen IgG-complexes through the amniotic fluid (for example, by aspiration or permeation through the fetal skin) [11, 12]. The baby was therefore fed with an amino acid formula diet until the age of 3 months. Surprisingly, at the age of 3 months, a challenge with cow’s milk formula was well tolerated, excluding CMP allergy as the cause for his eosinophilic proctocolitis. Hence, either he reacted to another food protein (egg or soy) through *in utero* sensitization, or maybe there was no food allergy at all, and we observed a “neonatal transient eosinophilic colitis".

Our case is the first newborn in Europe who fits the diagnosis of “neonatal transient eosinophilic colitis", presenting with hematochezia before the first feed, and with, upon investigation, blood eosinophilia as well as eosinophilic infiltrates in the gut tissue. So far, only two similar Japanese cases have been described, that is, newborns presenting with hematochezia before their first feed [13]. Blood analysis also showed marked eosinophilia and histopathology demonstrated diffuse massive eosinophilic infiltration of the lamina propria in both babies, who improved spontaneously after being exclusively parenterally fed during a few days, and tolerated normal breast milk later on.

Lymphoid hyperplasia within the gut as well as eosinophilia are two characteristic findings of hematochezia in early infancy, whether in FPIAP or neonatal transient eosinophilic colitis. Both entities present with “early” rectal bleeding and both involve eosinophils. We hypothesize that different chemical mediators are released in these entities, based on a different pathophysiological process. In fact, eosinophils can release several inflammatory mediators; for example, eosinophilic peroxidase, major basic protein, and leukotrienes. It is unclear at this stage which chemical mediator released by these eosinophils causes either a benign, transient inflammation *versus* FPIAP. Research into the transcriptome of mucosal biopsy specimens in babies is, however, promising, as it demonstrates an enhanced expression of the gene C-C Motif Chemokine Ligand 11 (*CCL11*; eotaxin-1) in very young neonates with FPIAP and gene C-X-C Motif Chemokine Ligand 13 (*CXCL13*) in older children with FPIAP [14–16]. The observation that *CCL11* is highly expressed in the gut of very young neonates is consistent with mucosal eosinophilia. Increased *CXCL13* expression is related to the generation of IgA, attracting B cells into developing lymphoid tissue.

## Conclusions

If a well-looking newborn baby presents with hematochezia before its first oral feed, FPIAP is a probable cause. The diagnosis of FPIAP needs to be confirmed by an abnormal oral challenge test once the hematochezia has disappeared. If such a challenge cannot demonstrate an allergic origin, as in our case, then the etiology of this hematochezia is not FPIAP but rather it is a neonatal transient eosinophilic colitis. We believe that neonatal transient eosinophilic colitis occurs more frequently than previously thought. Further research is needed to clarify the different pathophysiological roles of eosinophils in early hematochezia.

## Abbreviations

*CCL11*: Human gene encoding for the C-C motif chemokine 11 (= eosinophil chemotactic protein and eotaxin-1)
CMP: Cow’s milk protein
*CXCL13*: Human gene encoding for the chemokine ligand 13 (= B lymphocyte chemoattractant or B cell-attracting chemokine 1)
FPIAP: Food protein-induced allergic proctocolitis
Ig: Immunoglobulin
IL: Interleukin
INR: International normalized ratio
PCR: Polymerase chain reaction
RAST: Radioallergosorbent tests
TNF-alpha: Tumor necrosis factor-alpha.
